# Burnout in Israeli medical students: a national survey

**DOI:** 10.1186/s12909-023-04037-2

**Published:** 2023-01-24

**Authors:** Peter Gilbey, Mandy Moffat, Adi Sharabi-Nov, Omri Cohen, Galit Neufeld Kroszynski, Orit Karnieli-Miller, Roni Gillis, Jacob Urkin, Khen Moscovici

**Affiliations:** 1 The Azrieli Faculty of Medicine, Bar-Ilan University, 8 Henrietta Szold St, Safed, Israel; 2 Centre for Medical Education, University of Dundee, Dundee, UK; 3 Ziv Medical Center, Tel-Hai Academic College, Safed, Israel; 4 Department of Medical Education, Sackler Faculty of Medicine, Tel-Aviv University, Tel-Aviv, Israel; 5 Moshe Prywes Center for Medical Education, Faculty of Health Sciences, Ben-Gurion University of the Negev, Beersheba, Israel; 6 Shaare Zedek Medical Center, Jerusalem, Israel

**Keywords:** Burnout, Medical student, Undergraduate, Israel, Emotional Exhaustion, Cynicism

## Abstract

**Introduction:**

Professional burnout is characterized by loss of enthusiasm for work, cynicism, and a low sense of personal efficacy. Burnout may adversely affect medical professionalism. Burnout is common in clinicians and varying rates have been reported in medical students. No data exist regarding the prevalence of burnout among Israeli medical students. The aims of this study were to assess the rate of burnout in Israeli medical students and to identify students who were particularly susceptible to burnout.

**Methods:**

A cross-sectional questionnaire design was employed, gathering data from medical students in all years of study across three medical schools. Burnout was measured using the Maslach Burnout Inventory Student Survey (MBI-SS), translated into Hebrew.

**Results:**

Of the 2160 students in the participating medical schools, 966 (44.7%) completed MBI-SS and demographic questionnaires. The overall burnout rate was 50.6%. Multivariate logistic regression analysis yielded that female gender, age under 25, advanced year of study, studying at a specific medical school and not being a parent are all significantly correlated with higher levels of burnout.

**Conclusions:**

A high rate of burnout was found. The identification of young women who are not parents during advanced years of studies as being at-risk is important, in order to guide the development of burnout prevention interventions.

## Introduction

Professional burnout is characterized by a loss of enthusiasm for work, cynicism, and a low sense of personal accomplishment [1]. It involves a response to chronic interpersonal stressors experienced on the job [2]. Burnout may adversely affect medical professionalism and quality of care, increase the probability of medical errors, and lead to early retirement [3–6]. Burnout may also have severe personal implications, including alcohol abuse and suicidal ideation [7, 8]. A national survey of US physicians reported that 45.8% of physicians demonstrated at least one symptom of burnout in 2012, and 54.4% two years later [1, 9].

In Israel, levels of burnout up to 60% have been reported among practicing physicians [10–12]. Of all Israeli physicians, the highest levels of burnout have been found among interns and residents [13].

Although the exact prevalence varies amongst reported studies [14], burnout rates of up to 49.6% have been reported for medical students [15–17]. Different reports from around the world have found medical student burnout rates of 9.09–62% in Asia [18–21], 15.05–46% in Europe [22–26], 13–75% in the Middle East [27, 28], 49% in North America [29], 12–44.9% in South America [30–32], and 20% in New Zealand.

High rates of burnout can adversely impact students [33–36], the healthcare workforce [37] and ultimately patient outcomes [38–40].

Burnout and depressive symptoms have been found to predict serious thoughts of dropping out [37]. Burnout has been found to be inversely correlated with empathy [38, 39]. Alcohol abuse/dependence was found to be more common among students with burnout [36]. Regarding professionalism, students with burnout were significantly more likely to have copied during an examination or to have allowed other students to copy from them. They were also more likely to have reported a physical examination result as normal when it had actually been omitted from the examination, and to have said that they had ordered a test when they had not. Burnt out students held less altruistic views regarding doctors’ responsibility to society, and tended less to want to provide care to underserved populations [40]. Finally, academic burnout has been shown to adversely affect students’ learning [33].

Educators should understand the scale of the problem in their own contexts, and which factors are key modulators in burnout, in order to effectively reduce prevalence rates e.g. through appropriate curriculum design and student wellbeing services. Currently, no data exists regarding the rates of burnout in Israeli medical students.

Israeli basic medical training consists of two models: either a six-year undergraduate entry program, similar to the European model, or a four-year graduate-entry program, similar to that of the US [41, 42]. Another characteristic of Israeli medical students is that they are, on average, older that their international counterparts due to compulsory military service [42].

The aims of this study are to measure the rates of burnout in Israeli medical students and to identify variables that may increase the likelihood of burnout. There are conflicting reports regarding the populations most susceptible to medical student burnout. There seems to be little agreement regarding the age and gender of those most affected, the effect of financial burden, and the stage in medical training that is most problematic.

## Methods

A quantitative non-experimental cross-sectional design was employed with data collected via questionnaires.

All Israeli medical schools were invited to participate in the study to attempt to obtain a more representative national dataset. A local researcher in each medical school was approached to ensure access to the student body and to facilitate the successful completion of the study. All students currently enrolled in an Israeli MD programme were invited and were eligible to be included in the study. Students taking time out from study or on a placement outside Israel, were excluded.

The Maslach Burnout Inventory Student Survey (MBI-SS) was used to collect data. Its reliability and validity have been established [43, 44]. The MBI-SS consists of 15 questions targeting three dimensions of student burnout: emotional exhaustion, cynicism and lack of personal academic efficacy. All items are scored on a 7-point frequency rating scale ranging from 0: Never to 6: Always. Total possible scores range from 0 to 30 for the emotional exhaustion scale, 0 to 24 for the cynicism scale, and 0 to 36 for the personal efficacy scale [45].

The MBI-SS was translated from English into Hebrew by three native Hebrew speaking medical educators working independently of each other. Semantic differences in translations were discussed and a final common translation was agreed upon. Subsequently, back translation was done to English. The final questionnaire was checked by a native English- speaking medical educator who is also fluent in Hebrew.

In the MBI-SS there is no universal consensus as to cut-off points for the definition of burnout [14]. It has been suggested that burnout is primarily a two-dimensional construct with an effective component (emotional exhaustion) and an attitudinal component (cynicism) [44]. Some authors have used a “liberal two-dimensional” construct with high emotional exhaustion or high cynicism [16, 46]. Others suggested a “two-dimensional burnout”, requiring high scores both on emotional exhaustion and cynicism [32]. The adoption of the more liberal “two-dimensional” construct, requiring a high score on either emotional exhaustion or cynicism, has the limitation of possibly over-estimating burnout, although it has been claimed that this construct best reflects burnout, and that anyone with either high emotional exhaustion or high cynicism could indeed be defined as burnt-out and in need of help. In addition, this is the construct most commonly used in the definition of physician burnout, and therefore the use of this construct would enable comparison of the results of this study with the results of other studies, historically and in the future. However, in order not to over-estimate burnout, for the purpose of this study, medical student burnout was defined as two-dimensional burnout, with scores of 15 or higher on the emotional exhaustion scale and 7 or higher on the cynicism scale [32].

A demographic and personal questionnaire was developed and included in the study. To explore financial stress [47, 48], the demographic questionnaire included factors deemed to be relevant to students’ financial burden. These data included the monetary support received from parents and the source of payment of tuition fees.

Ethics review board approval was obtained from all three participating medical schools, one in the North of Israel, one in the centre, and one in the South. All data collection was completed by the end of 2019, prior to the outbreak of the COVID-19 pandemic.

In the participating medical schools, questionnaires were initially distributed to all Israeli medical students, from all years of study by local researchers via email. Reminder emails were sent out two weeks later, with the assistance of student bodies. Following this, student research assistants distributed paper questionnaires, mainly to pre-clinical students who tended to be more available in large-group teaching settings. Students answering on-site were specifically instructed not to respond to the questionnaire if it had been previously answered online. The survey was accompanied by a letter explaining the nature and the general aim of the study and emphasizing the anonymity of the participants. Both electronic and paper responses were entered into Excel and then into SPSS.

### Statistical analysis

Pearson’s chi-squared were applied for testing the correlations between the different medical schools and socio-demographics and personal characteristics. Due to the large sample, and according to the central limit theorem, normality of the continuous variables was assumed. Cronbach’s alpha coefficients were calculated to assess the internal consistency for each subscale of the MBI-SS questionnaire. Multivariate analysis of variance (MANOVA) tests were applied to measure the differences between the three medical schools and other socio-demographic and personal characteristics for the burnout sub-scales using Wilks’s statistics. Univariate logistic regressions were applied for testing the correlations between the burnout groups and demographic variables, provided as ORs and 95% confidence interval (CI). A multiple logistic regression model was applied in order to test the correlations between the two examined groups of burnout and socio-demographic variables using only significant variables from the univariate logistic regressions. A *p*-value of 5% or less was considered statistically significant. The data were analysed using SPSS version 25 (SPSS Inc., Chicago, IL, USA).

## Results

Of the 2160 students in the participating faculties who were invited to participate, 966 (44.7%) completed MBI-SS and demographic questionnaires. No information was available regarding the characteristics of non-participating students. The demographic characteristics of all responders are presented in Table 1. Cronbach’s alphas were calculated for each of the MBI-SS subscales and these fell in the acceptable range (0.7–0.8) [49] and can be seen in Table 2. Burnout rates for the main sub-scales of the MBI-SS are presented in Table 3.

**Table 1:** Demographic characteristics of the study participants according to medical school

Variables\Medical School	All participant (*n* = 966)	Ben-Gurion (*n* = 515)	Tel-Aviv (*n* = 232)	Bar-Ilan (*n* = 219)	p
Response rate (%)	44.7	71.5	24.2	45.6	
Gender (n, %)					
Female	575, 59.5	313, 60.8	140, 60.3	122, 55.7	0. 422
Male	391, 40.5	202, 39.2	92, 39.7	97, 44.3	
Age group (n, %)					
< 25	317, 32.8	201, 39.0	87, 37.5	29, 13.2	< 0.001
26–30	545, 56.4	284, 55.1	122, 52.6	139, 63.5	
31–40	104, 10.8	23, 9.9	23, 9.9	51, 23.3	
Ethnicity (n, %)					
Jewish	875, 90.7	468, 91.1	209, 90.1	198, 90.4	0.905
Other	90, 9.3	46, 8.9	23, 9.9	21, 9.6	
Country of birth (n, %)					
Israel	877, 90.8	473, 91.9	208, 89.7	196, 89.5	0.174
Soviet Union	38, 3.9	14, 2.7	10, 4.3	14, 6.4	
Other	51, 5.3	28, 5.4	14, 6.0	9, 4.1	
Family status (n, %)					
Bachelor	356, 36.9	194, 37.7	79, 34.1	83, 37.9	0.009
Married	239, 24.7	112, 21.7	56, 24.1	71, 32.4	
Living with a partner	371, 38.4	209, 40.6	97, 41.8	65, 29.7	
Parent of child/children? (n, %)					
No	866, 90.0	470, 92.0	208, 89.7	188, 85.8	0.040
Yes	96, 10.0	41, 8.0	24, 10.3	31, 14.2	
Year of study (n, %)					
1^st^	284, 29.4	142, 27.6	47, 20.3	95, 43.4	< 0.001
2^nd^	179, 18.5	9, 1.7	77, 33.2	93, 42.5	
3^rd^	205, 21.2	148, 28.7	39, 16.8	18, 8.2	
4^th^	129, 13.4	70, 13.6	46, 19.8	13, 5.9	
5/6^th^	169, 17.5	146, 28.4	23, 9.9	0, 0

**Table 2:** Cronbach’s alpha of the MBI-SS subscales according to country and year of study (including current study)

Country	Name of 1^st^ author	Sample size	Year	MBI-SS subscales		
				Emotional Exhaustion	Cynicism	Academic Efficacy
Saudi Arabia	Almalki	249	2017	0.85	0.76	0.75
Serbia	Ilic	760	2017	0.87	0.86	0.85
Brazil	Dos Santos Boni	265	2018	0.80	0.83	0.74
China	Liu	453	2018	0.89	0.90	0.85
Sri Lanka	Wickramasinghe	194	2018	0.84	0.87	0.88
Germany	Erschens	597	2018		0.81–0.86	
**Israel**	**Gilbey (Current study)**	966	2023	0.78	0.87	0.71

**Table 3:** Burnout in Israeli Medical students according to main MBI-SS subscales

MBI-SS		Not burnt out (less than cut-off point)		Burnt out (above cut-off point)	
Subscales	Cut-off point	n	%	n	%
Emotional Exhaustion	15	286	29.6	680	70.4
Cynicism	7	414	42.9	552	57.1

Using the established cut-off points, 223 students (23.1%) did not demonstrate any type of burnout, 254 students (26.3%) demonstrated one burnout factor (uni-dimensional burnout), 489 students (50.6%) demonstrated two factors of burnout (two-dimensional burnout), and 743 (76.9%) demonstrated at least one factor of burnout (liberal two-dimensional burnout).

For the purpose of both univariate and multivariate logistic regressions, we used the group of students showing no burnout and the group of students showing two-dimensional burnout, in order to accentuate the differences.

**Fig. 1:**
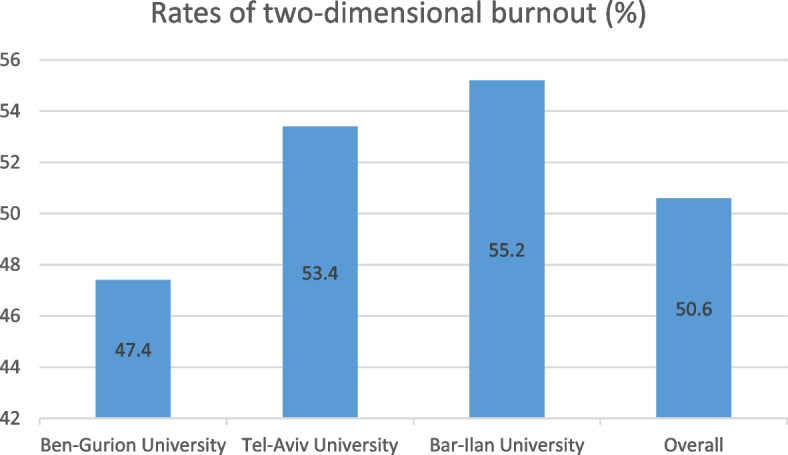
Rates of two-dimensional burnout

The overall rates of two-dimensional burnout are presented in Fig. 1.

Significant differences were found between the different medical schools on the MBI-SS subscales, (F_(6,1922)_ = 3.74, *p* = 0.001, Eta^2^ = 0.001). For the Cynicism subscale, the mean score of the Tel-Aviv medical school was significantly higher than the Ben-Gurion medical school (M-9.5, standard deviation (SD) 6.4 vs M-8.3, SD 5.9, *p* = 0.05). For the Academic Efficacy subscale, the mean score of the Tel-Aviv medical school was significantly lower than the Bar-Ilan medical school (M-20.3, SD 6.3 vs M-22.0, SD 6.2, *p* = 0.008).

A significant effect of the student being a parent on the MBI-SS subscales was found only for the Emotional Exhaustion subscale (F_(3,958)_ = 3.81, *p* = 0.010, Eta^2^ = 0.012). Emotional Exhaustion was significantly higher among non-parent students compared to parent students (M-18.8, SD 7.4 vs M-17.1, SD 7.3, *p* = 0.035).

A significant effect of gender on the MBI-SS subscales was found for the Emotional Exhaustion and Academic Efficacy subscales (F_(3,962)_ = 14.36, *p* < 0.001, Eta^2^ = 0.043). Female students showed significantly higher Emotional Exhaustion (M-19.5, SD 6.3 vs M-17.2, SD 8.6, *p* < 0.001), and significantly lower Academic Efficacy (M-20.9, SD 5.9 vs M-21.9, SD 6.1, *p* = 0.009), when compared to male students.

A significant effect of the student having a parent who is a physician on the MBI-SS Emotional Exhaustion subscale was found (F_(3,962)_ = 2.66, *p* = 0.047, Eta^2^ = 0.01). Emotional Exhaustion was significantly lower among those students that have parent/s who are physicians (M-17.5, SD 7.1 vs M-18.9, SD 7.5, *p* = 0.024). The other two subscales did not show any significant differences between the two groups.

A significant effect of monthly monetary support from parents on the MBI-SS Emotional Exhaustion subscale was found (F_(6,1922)_ = 2.16, *p* = 0.044, Eta^2^ = 0.01). Students who received less than 625 USD per month (partially supported) showed significantly lower sub-scale scores compared to students who did not receive any parental support or those receiving more than 625 USD per month (M-17.9, SD 7.1 vs M-18.9, SD 8.7 vs M-19.2, SD 6.7, respectively, *p* = 0.05).

A significant effect of year of study on the MBI-SS subscales, (F_(9,2336)_ = 7.96, *p* < 0.001, Eta^2^ = 0.024) was found. The mean score of the Emotional Exhaustion sub-scale during the first and second years of study was significantly lower compared to the other years of study (M-17.0, SD 7.0 vs M-19.8–20.5, SD 6.4–8.7, *p* < 0.001). At the first, second and third years of study the mean scores of the Cynicism subscale were significantly lower compared to the fourth, fifth and sixth years of study (clinical years) (M-7.4–8.8, SD 5.9–6.1 vs M-10.5–10.7, SD 6.5, *p* < 0.001). The highest Academic Efficacy sub-scale scores were measured at the first and second years while the lowest were measured at the fifth and sixth years of study (M-22.1, SD 6.2 vs M-20.0–21.1, SD 5.5–5.9, *p* < 0.001).

A significant effect of age group on the MBI-SS subscales, (F_(6,1922)_ = 2.78, *p* < 0.05, Eta^2^ = 0.010) was found. Cynicism was significantly lower in the youngest age group (< 25 years old) compared to the middle age group (26–30 years old) (M-7.4, SD 6.0 vs M-9.2, SD 6.3, *p* = 0.008). The highest Academic Efficacy was measured in the youngest age group while the lowest was measured in the oldest age group (30 + years old) (M-22.1, SD 5.8 vs M-20.7, SD 6.9, *p* = 0.012).

No effect of the country of birth on burnout was found, and rates of burnout in first-generation immigrants were not significantly different from Israeli-born students. No effect of the source of tuition fees payment on burnout was found.

Univariate logistic regression analysis yielded that gender, age, year of study, medical school and parental status are the socio-demographic variables that are significantly correlated with levels of burnout. The student having a physician parent or parents, source of tuition payment, family status and monthly monetary support from parents were not found to be significantly correlated with burnout levels. Female students were burned out 1.84 times more compared to the male students (95% CI 1.31–2.59). Students in the 26–30 years old age group were 50% more burned out compared to students younger than 25 years old (95% CI 1.05–2.20), while students older than 30 years old showed the same level of burnout as the students younger than 25. Compared to the 1st and 2nd years of study, students in their 3rd year are burned out twice as much (OR = 2.07, 95% CI 1.35–3.15), whereas students at their 4th and 5th/6th year of study are at least three times more burnt out (OR = 3.83, 95% CI 1.66–8.86 and OR = 3.24, 95% CI 1.83–5.74 respectively). Students from the Ben-Gurion and Tel-Aviv faculties are 50% and 20% respectively less burned out compared to the Bar-Ilan students (95% CI 0.31–0.74 and 95% CI 0.50–1.30 respectively) see Table 4.

**Table 4:** Univariate logistic regression analysis for the correlation between variables and level of burnout

Variable	Type of variable	Category	OR	95%CI	p
Gender	Binary	Male	1.00	Reference	
		Female	2.15	1.56–2.96	< 0.001
Age group (years)	Ordinal	< 25	1.00	Reference	
		26–30	1.41	0.99–2.00	0.055
		31–40	0.90	0.53–1.52	0.690
		P for trend			0.066
Year of study	Ordinal	1^st^/ 2^nd^	1.00	Reference	
		3^rd^	2.00	1.31–3.05	0.001
		4^th^	3.35	1.90–5.91	< 0.001
		5^th^/6^th^	2.91	1.80–4.72	< 0.001
		P for trend			< 0.001
Medical school	Nominal	Bar Ilan	1.00	Reference	
		Ben-Gurion	0.61	0.40–0.93	0.020
		Tel-Aviv	0.80	0.50–1.30	0.376
Is the student a parent?	Binary	No	1.00	Reference	
		Yes	0.59	0.36–0.98	0.040
Does the student have a physician parent	Binary	No	1.00	Reference	
		Yes	0.81	0.54–1.21	0.302
Source of tuition	Binary	Myself	1.00	Reference	
Payment		Parents/Other	1.09	0.76–1.57	0.638
Family status	Binary	Married/Living with a partner	1.00	Reference	
		Bachelor	1.24	0.89–1.73	0.199
Monthly monetary	Ordinal	No	1.00	Reference	
Support by parents		Less than $625	0.95	0.64–1.41	0.801
		More than $625	1.31	0.86–1.99	0.211
		P for trend			0.221

*OR* Odds ratios, *CI* Confidence intervals

A multivariate logistic regression was carried out to evaluate the correlation between the levels of burnout and all significant socio-demographic variables from the univariate logistic regression. Female students were burned out 1.87 times more compared to the male students (95% CI 1.28–2.74). Levels of burnout were positively and significantly correlated with year of study (*p* for trend < 0.001). Compared to the 1st and 2nd years of study, students in their 3rd year are burned out almost 5 times more (OR = 4.81, 95% CI 2.82–8.22), students in their 4th year more than 3 times more and students in their 5th/6th year almost ten times more (OR = 3.84, 95% CI 1.56–9.47 and OR = 9.90, 95% CI 4.87–20.16 respectively).

Levels of burnout were negatively and significantly correlated with age of students (*p* for trend < 0.001). Students in age group of 26–30 years old were 25% less burned out compared to students younger than 25 years old (95% CI 0.47–1.18) and students older than 30 years old were 63% less burnout compared to the youngest students (95% CI 0.18–0.75).

Students from Ben-Gurion and Tel-Aviv faculties are 86% and 60% less burned out, respectively, compared to the Bar-Ilan students (95% CI 0.07–0.25 and 95% CI 0.23–0.71 respectively) see Table 5.

**Table 5:** Multivariate logistic regression models for the correlation between significant variables and level of burnout

Variable	Type of variable	Category	OR	95%CI	p
Gender	Binary	Male	1.00	Reference	
		Female	2.16	1.51–3.08	< 0.001
Age group (years)	Ordinal	< 25	1.00	Reference	
		26–30	0.75	0.48–1.17	0.198
		31–40	0.50	0.25–1.03	0.061
		P for trend			< 0.001
Year of study	Ordinal	1^st^/ 2^nd^	1.00	Reference	
		3^rd^	4.22	2.51–7.08	< 0.001
		4^th^	6.02	3.15–11.52	0.001
		5/6^th^	8.69	4.64–16.30	< 0.001
		P for trend			< 0.001
Medical school	Nominal	Bar Ilan	1.00	Reference	
		Ben-Gurion	0.16	0.09–0.29	0.001
		Tel-Aviv	0.39	0.23–0.69	0.001
Is the student a parent?	Nominal	No	1.00	Reference	
		Yes	0.52	0.29–0.59	0.034

*OR* Odds ratio, *CI* Confidence interval

## Discussion

The aims of this large, multi-school study were to examine the rate of burnout in medical students in Israel, and to explore whether certain variables were more likely to put students at risk of burnout. Thus, effective interventions could be preferentially implemented [50]. We found an overall prevalence of burnout in Israeli Medical students of 50.6% according to the defined two-dimensional cut-off criteria.

When compared to the pooled data from a recent meta-analysis [14], Israeli students demonstrate lower scores on Emotional Exhaustion and Cynicism, the two primary components of burnout (indicating less burnout), although they did demonstrate lower levels of Academic Efficacy (indicating more burnout). This could be attributed to the fact that following a compulsory military service of between two and three years, the Israeli students were older in comparison to other students around the world. The range of the mean ages of the students in the studies included in the meta-analysis was 19.23–29.38 years [14], whereas in this current study only 32.8% of participants were 25 years old or younger, and 67.2% were 26 years old or older. In this study, multivariate logistic regression has shown that levels of burnout were negatively and significantly correlated with the age of the students, with the highest levels of burnout in the youngest age group.

This study also attempted to identify populations particularly susceptible to burnout in order to guide early identification and intervention efforts. There was a significant difference in burnout rates found between the medical schools included in this study. The overall two-dimensional burnout rates tended to be lower in the Ben-Gurion University in comparison to Tel-Aviv University and Bar-Ilan University, although these differences were not significant. Examination of the sub-scale scores of the MBI-SS reveals that Cynicism was significantly higher in Tel-Aviv than in Ben-Gurion and Bar-Ilan, and Academic Efficacy was higher (indicating less burnout) in Bar-Ilan than in Tel-Aviv. The reason for these differences is unclear. Differences in burnout rates could be due to differences in curricula and academic load [51, 52]. The Bar-Ilan and Tel-Aviv Universities both run 4-year programmes, whereas the Ben-Gurion University only runs a six-year programme. Academic load is greater in a 4-year programme.

Another explanation may be related to the time of year that data was collected. Due to the different times of ethics approval, there was a seasonal difference in the times that the questionnaires were distributed in the participating medical schools. Data collection started in January (before the first semester exams), March (mid-year, immediately after the first semester exams) and September (toward the end of the summer break) in the Bar-Ilan, Ben-Gurion and Tel-Aviv Universities, respectively. This may also have had an effect on burnout rates. Further work is required to understand why these differences in burnout scores between medical schools exist.

No significant differences were found between students born in Israel and those who were born outside of Israel. This is in contrast to the study by El-bar et al. (2013) who found that ‘immigration to Israel’ was a significant factor in reporting of compassion fatigue, which has previously been described as a form of burnout, in a group of family practitioners [10]. This is possibly due to the fact that the significant wave of immigration to Israel was from the former Soviet Union in the early 1990s, and most of the participating students would have immigrated at a very early age, and most of the trauma of immigration would have been experienced by their parents.

In this study, no effect of the source of payment of tuition fees on burnout was seen. This is in contrast to other studies that found that financial concerns had significant negative mental health consequences for students. Financial responsibilities were among the stressors rated highest by students [52], and having significant financial debt was significantly associated with suicidal ideation during the previous year [16]. Financial concerns have also been shown to be a risk factor for burnout [47, 48], and students who were employed (probably indicating greater financial stress) were twice as likely to suffer from burnout when compared with non-employed students [48]. On the other hand, a multi-institutional study of burnout in US medical students found that although financial stress predicted burnout, the amount of debt was not found to predict burnout [48]. Annual tuition fees in Israel are currently the equivalent of approximately 3200 USD.

In this study, Emotional Exhaustion was significantly lower among those students that have parent/s who are physicians. Having a parent/parents who are physicians could potentially be a financial and academic support system that could reduce stress and burnout.

For the purposes of this study, monetary support from parents was divided into three categories: No support, up to 625 USD, and more than 625 USD. These amounts were considered by the local students’ associations to be significant cut-off points in the current financial reality in Israel. A significant effect of monthly monetary support from parents on the MBI-SS was found for Emotional Exhaustion. The group receiving the most support seem to be the most burnt out. The meaning of this remains unclear. This may be related to a culture that encourages family relationships and collectivism while expecting individualism and self-support. Bar-Ilan University medical students were significantly more likely to be financially supported by parents in comparison to students in other faculties. This could potentially be related to the fact that Bar-Ilan students also tended to be older, married and parents of children.

The results of this study indicate that Emotional Exhaustion is significantly lower in students who are parents. This is in contrast to the hypothesis that an objective lack of free time would lead to higher rates of burnout, but in agreement with a multi-institutional study of burnout in US medical students that found that parenthood was not found to predict burnout [48]. This may be related to the Israeli culture that encourages childbirth and connects parenthood with fulfilment.

This study shows that female medical students consistently demonstrate higher levels of burnout than their male counterparts. We found significantly higher Emotional Exhaustion and significantly lower Academic Efficacy among female students. Previous research has reported mixed results. For example, Dyrbye et al. (2010) found that gender did not predict burnout [48]. However, in preclinical medical students in Beirut, Lebanon, it was found that being female was correlated with higher stress and burnout [28]. Female pre-medical students in San-Diego, US have been shown to have especially high rates of burnout [45]. Interestingly, unmarried females felt more burnout, possibly due to concern with the future of balancing between career and family life.

This study indicates that burnout on all three subscales is lowest during the first years of study, and it increases with students’ progress through medical school, into their clinical years. This finding is compatible with previous studies that showed that empathy significantly declines during the third year of medical school, after entry into the clinical environment [53].The factors most strongly related to burnout among clinical students were general dissatisfaction with the overall learning environment, clerkship organization and cynical residents [54]. A study of pre-clinical and clinical medical students in Ireland found that burnout was significantly higher in the clinical years [23]. Most incidents of medical student abuse were reported to occur during clinical rotations [55], possibly contributing to the raised levels of burnout reported during the clinical years. The hidden curriculum, that is encountered on a daily basis during clinical rotations, can encourage cynicism and promote personal distress and burnout [56]. However, a multi-institutional study of burnout in US medical students found that year in school was not found to predict burnout [48]. In summary, evidence shows different rates of burnout during progression through medical school. Some studies have reported that burnout increases with each year of study, whereas others have found that burnout is highest in the third year of study [14].

In this study, burnout was found to be highest in the youngest students. As previously mentioned, Israeli medical students are much older in general than their international counterparts. A multi-institutional study of burnout in US medical students found that demographic characteristics were not associated with burnout, and age was not found to predict burnout [48].

This study has several possible limitations. Although originally intended to be a comprehensive national survey, technical and procedural difficulties prevented the participation of students from two Israeli medical faculties (The Hebrew University Hadassah Medical School in Jerusalem and The Ruth and Bruce Rappaport Faculty of Medicine at the Israel Institute of Technology (Technion), and a third (The Adelson school of medicine at the Ariel university) was excluded as it only opened its first year in October 2019. The rates of burnout in the non-participating schools are unknown. In addition, the relatively low response rate in The Tel-Aviv University School of Medicine may mean that the results are less representative of this school. The effect of burnout on response rates is unknown [16]. Due to the data collection methods, the sample was slightly biased toward pre-clinical students. The level of burnout among non-responders is unknown. Furthermore, the selection of the source of tuition fee payment may not have been the ideal choice to indicate financial distress.

Further studies should attempt to include all Israeli medical schools. Other instruments should be used to assess symptoms of depression and measure quality of life, and examine the correlation between these parameters and burnout. Longitudinal quantitative studies following the same cohort of students over time could elucidate the change in burnout rates with progression through medical school. Qualitative studies should be conducted in order to explore the students' experiences of burnout and its sources in depth.

## Conclusion

In summary, the results of this study indicate a high rate of burnout among Israeli medical students. These results are compatible with the results of other studies from around the world examining medical student burnout. Female gender, younger age, advanced year of study, attending a specific medical school, and not being a parent were all associated with higher levels of burnout. The results of this study should form the basis for the emerging discussion on medical student well-being in Israel, and guide the development of burnout prevention interventions.

### Practice points

- Burnout is common in clinicians and varying rates have been reported in medical students.
- No data currently exist regarding the prevalence of burnout among Israeli medical students and correlation with relevant demographic characteristics.
- The aims of this cross-sectional questionnaire study were to assess the rate of burnout in Israeli medical students in all years of study using the MBI-SS and to identify students particularly susceptible to burnout.
- The results of this study indicate a high rate of burnout among Israeli medical students
- Female gender, younger age, advanced year of study, attending a specific medical school, and not being a parent were all associated with higher levels of burnout

## Data Availability

The datasets used and analysed during the current study are available from the corresponding author on reasonable request.
